# miR-19b-3p relieves intervertebral disc degeneration through modulating PTEN/PI3K/Akt/mTOR signaling pathway

**DOI:** 10.18632/aging.203553

**Published:** 2021-09-23

**Authors:** Yulin Zhao, Aimin Li

**Affiliations:** 1 Department of Spine Surgery, Qilu Hospital (Qingdao), Cheeloo College of Medicine, ShanDong University, Qingdao 266035, ShanDong, China

**Keywords:** intervertebral disc degeneration, miR-19b-3p, PTEN, apoptosis, signaling pathways

## Abstract

Emerging studies have revealed that non-coding RNAs contribute to regulating intervertebral disc degeneration (IVDD). Here, we intended to probe into the function of *miR-19b-3p* in IVDD evolvement. The *miR-19b-3p* level in the intervertebral disc (IVD) tissues of IVDD patients and IL-1β/TNF-α/hydrogen peroxide-treated human nucleus pulposus cells (HNPCs) was determined by quantitative real-time polymerase chain reaction (qRT-PCR). Also, qRT-PCR was conducted to examine the profiles of MMP-3, MMP-9, MMP-13, ADAMTS-4 and ADAMTS-5. The PTEN/PI3K/Akt/mTOR pathway was examined by Western blot (WB). The *miR-19b-3p* overexpression assay was carried out, and HNPC proliferation and apoptosis were compared by the cell counting kit-8 (CCK-8) assay and flow cytometry (FCM). In addition, the mechanism of action of *miR-19b-3p* was clarified using the PTEN inhibitor (VO-Ohpic triphosphate) or the mTOR inhibitor (Rapamycin) on the basis of IL-1β intervention and *miR-19b-3p* mimics transfection. Our results testified that *miR-19b-3p* expression was curbed in IVD tissues of the IVDD patients (vs. normal IVD tissues) and IL-1β-, TNF-α, or hydrogen peroxide-treated HNPCs. Up-regulating *miR-19b-3p* enhanced HNPC proliferation and hampered its apoptosis. Moreover, *miR-19b-3p* dampened the PTEN profile and activated the PI3K/Akt/mTOR pathway. Interestingly, attenuating PTEN reduced IL-1β-, TNF-α-, or hydrogen peroxide-mediated HNPC apoptosis and up-regulated PI3K/Akt/mTOR, while inhibiting the mTOR pathway offset the protective function of *miR-19b-3p*. Further mechanism studies illustrated that *miR-19b-3p* targeted the 3’untranslated region (UTR) of PTEN and abated the PTEN level. This research confirmed that *miR-19b-3p* suppressed HNPC apoptosis in the *in-vitro* model of IVDD by regulating PTEN/PI3K/Akt/mTOR pathway.

## INTRODUCTION

The intervertebral disc (IVD) is a moderately moving joint that provides load transfer and flexibility to the spine [1]. On the other hand, intervertebral disc degeneration (IVDD) is a chronic progressive process accompanied by loss of living cells in IVD, especially in the internal region of the nucleus pulposus (NP), and the imbalance of extracellular matrix (ECM) decomposition and catabolism [2, 3]. IVDD often causes low back pain and radiating leg pain in patients. Discogenic low back pain (DLBP) is the main risk factor of disability, and inflammation is considered to be the main driver of IVDD [4, 5]. Disappointingly, the treatment of IVDD does not fully restore the biological function of IVD, so there is still a need to explore the relevant mechanisms to bring novel theoretical guidelines for the clinical treatment of IVDD.

*miR-19b-3p* is a non-coding RNA that has been exhibited to contribute to cell inflammation and cell growth. Studies have revealed that pathologic circulatory stretch enhances vascular smooth muscle cell (VSMC) proliferation by curbing the *miR-19b-3p*/connective tissue growth factor (CTGF) pathway, thus inducing vascular remodeling [6]. On the other hand, several reports have confirmed the value of *miR-19b-3p* in orthopedic diseases. For example, *miR-19b-3p* boosts the proliferation and osteogenic differentiation of bone marrow mesenchymal stem cells (BMSCs) by interacting with *lncRNA H19*, thereby hindering postmenopausal osteoporosis (OP) [7]. Some reports have stated that *miR-19b-3p* attenuates OA evolvement by targeting EZH2 [8]. In another study, *miR-19b-3p* impedes GRK6 expression in a targeted manner, thereby attenuating IL-1β-induced cartilage extracellular matrix degradation and inflammation [9]. Overall, *miR-19b-3p* exerts an essential function in regulating cell inflammation, while its role in IVDD remains elusive.

In recent years, the PTEN/PI3K/Akt signaling pathway has attracted wide attention on account of its regulation on cells. Meanwhile, multiple reports have proved that PTEN/PI3K/Akt advances the growth of various cancers, such as osteosarcoma [10], invasive gallbladder adenocarcinoma [11] and non-small cell lung cancer [12]. In contrast, in the non-tumor field, some researchers have found that arctiin chokes high glucose-mediated proliferation of human retinal capillary endothelial cells (HRCECs) by activating ROCK1 and PTEN and inactivating PI3K and Akt [13]. Moreover, *miR-140-3p* heightens C2C12 cell proliferation and differentiation and hampers apoptosis by targeting PTEN and choking PTEN/PI3K/Akt in OP [14]. Notably, PTEN has been validated to regulate the behaviors of NP cells by directly targeting PI3K/AKT [15]. As a classical signaling pathway, PTEN/PI3K/Akt’s interaction with *miR-19b-3p* in IVDD remains largely unknown.

The mTOR pathway boosts metabolism, contributes to cell apoptosis and autophagy, and exerts a considerable role in diversified diseases. For example, Zheng RH et al. have stated that liraglutide chokes myocardial fibrosis and dysfunction by abating the mTOR/p70S6K signaling and enhancing autophagy [16]. In addition, several researchers have found through *in vitro* experiments in rats that hUMSCs dampen the autophagy of interstitial cells (ICs) by reducing oxidative stress and regulating the AMPK/mTOR pathway, thereby decreasing the apoptosis of ICs [17]. It is worth noting that *miR-21* facilitates the catabolism of type II collagen (Col II) and aggrecan by repressing the PTEN/Akt/mTOR signal autophagy in HNPCs [18].

Overall, this research focuses on probing the regulatory role and mechanism of *miR-19b-3p* on IVDD. We set up *in-vitro* IVDD models by treating HNPCs with IL-1β, TNF-α, or hydrogen peroxide. Our results manifested *miR-19b-3p* expression was impeded in IVDD tissues and HNPCs treated with IL-1β, TNF-α, or hydrogen peroxide separately. Further investigations suggested that overexpressing *miR-19b-3p* attenuated the IL-1β-mediated apoptosis of HNPCs and motivated the PI3K/Akt/mTOR pathway. These data hinted that *miR-19b-3p* and the PI3K/Akt/mTOR pathway are essential therapeutic targets for IVDD.

## RESULTS

### *miR-19b-3p* was notably down-regulated in IVD tissues of IVDD patients

**Figure 1:**
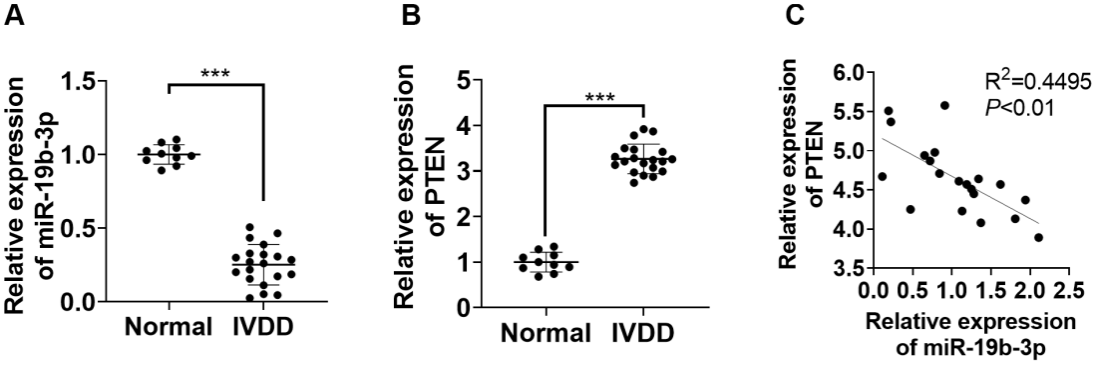
***miR-19b-3p* expression was hampered in the IVD tissues of IVDD patients.** (**A**, **B**) The *miR-19b-3p* and PTEN profiles in the IVD tissues of 20 IVDD patients and 10 normal IVD tissues were gauged by qRT-PCR. (**C**) Pearson was adopted to ascertain the link between *miR-19b-3p* and PTEN in the IVD tissues of IVDD patients. *** *P* <0.001(vs. normal group).

First, we performed qRT-PCR to monitor the *miR-19b-3p* and PTEN profiles in the IVD tissues of IVDD patients. According to the results, by contrast with that in normal IVD tissues, the *miR-19b-3p* level was attenuated (*P*<0.05, Figure 1A), while PTEN was up-regulated in the IVD tissues of IVDD patients (*P*<0.05, Figure 1B). Furthermore, Pearson analysis confirmed that the expression of *miR-19b-3p* and PTEN was reversely associated (R^2^=0.4495, *P*<0.01, Figure 1C). These outcomes manifested that *miR-19b-3p* influenced IVDD by regulating PTEN.

### *miR-19b-3p* expression was curbed in IL-1β-, TNF-α-, or hydrogen peroxide-treated HNPCs

**Figure 2:**
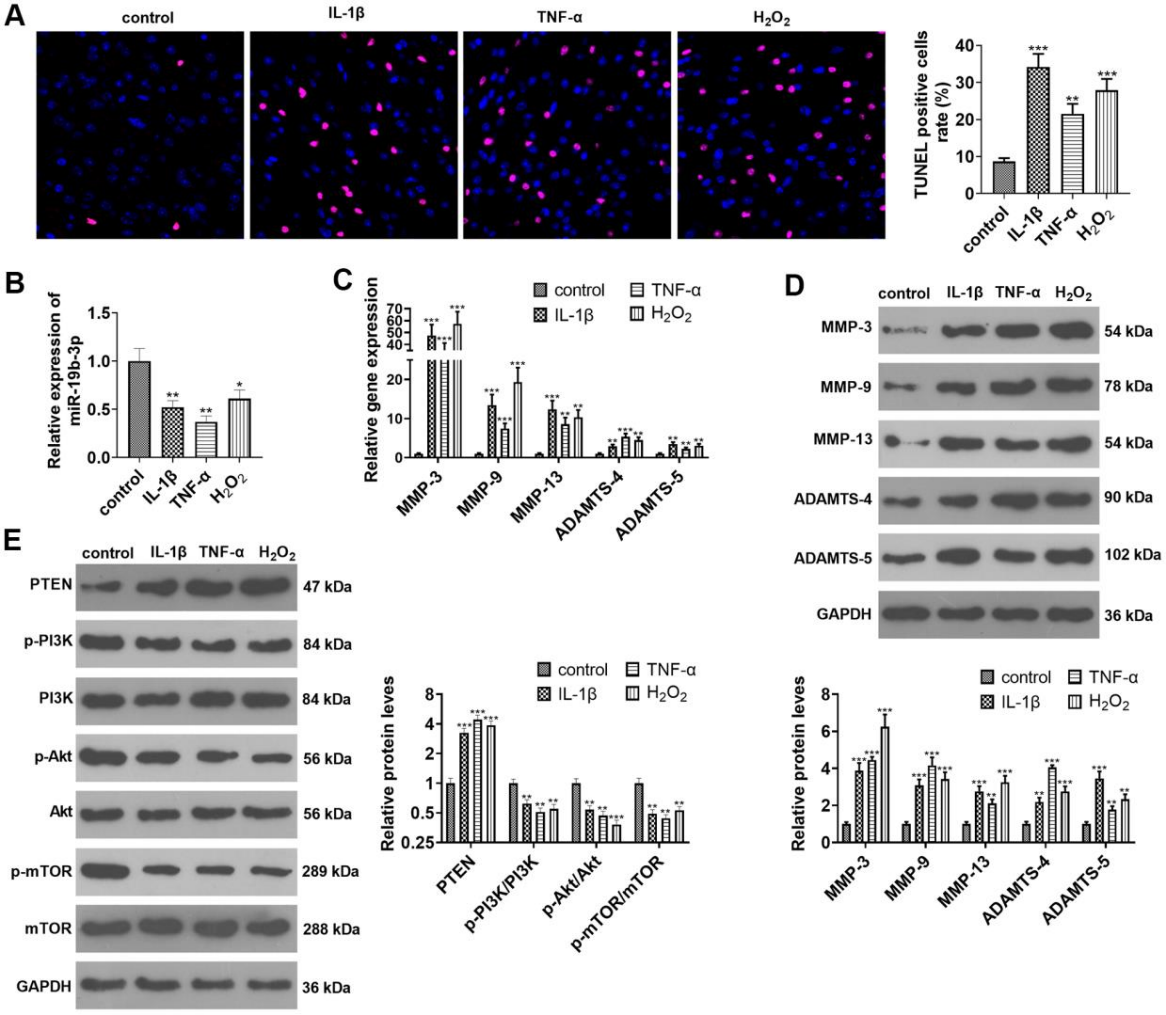
***miR-19b-3p* expression was impeded in IL-1β/TNF-α/hydrogen peroxide-treated HNPCs.** HNPCs were treated with IL-1β (10 ng/mL), TNF-α (40 ng/mL) and hydrogen peroxide (500 μM) for 24 hours to establish an *in vitro* IVDD model. (**A**) TUNEL staining was employed for apoptosis detection. (**B**) The *miR-19b-3p* level was testified by qRT-PCR. (**C**, **D**) The profiles of matrix metalloproteinases MMP-3, MMP-9, MMP-13, ADAMTS-4 and ADAMTS-5 were compared by qRT-PCR and WB. (**E**) The PTEN/PI3K/Akt/mTOR profile was verified by WB. * *P* <0.05, ** *P* <0.01, *** *P* <0.001(vs. control group). N=3.

HNPCs were treated with IL-1β, TNF-α or hydrogen peroxide separately. TUNEL staining exhibited that IL-1β or TNF-α or hydrogen peroxide treatment amplified the percentage of TUNEL-positive cells (vs. the control group) (*P*<0.05, Figure 2A). As indicated by qRT-PCR data, the treatment of IL-1β, TNF-α, or hydrogen peroxide down-regulated *miR-19b-3p* (vs. the control group) (*P*<0.05, Figure 2B). As displayed in Figures 2C and D, IL-1β, TNF-α and hydrogen peroxide all heightened the expression of MMP-3, MMP-9, MMP-13, ADAMTS-4 and ADAMTS-5, as confirmed by qRT-PCR and WB (*P*<0.05). Besides, WB outcomes validated that treatment of HNPCs with IL-1β, TNF-α, or hydrogen peroxide brought about a substantial elevation in PTEN expression and a decrease in p-PI3K/PI3K, p-Akt/Akt, and p-mTOR/mTOR expressions (*P*<0.05 vs. control group, Figure 2E). These results illustrated that IL-1β, TNF-α or hydrogen peroxide intensified apoptosis and down-regulated *miR-19b-3p* expression in HNPCs.

### Overexpressing *miR-19b-3p* weakened IL-1β-, TNF-α-, or hydrogen peroxide-mediated HNPC apoptosis

**Figure 3:**
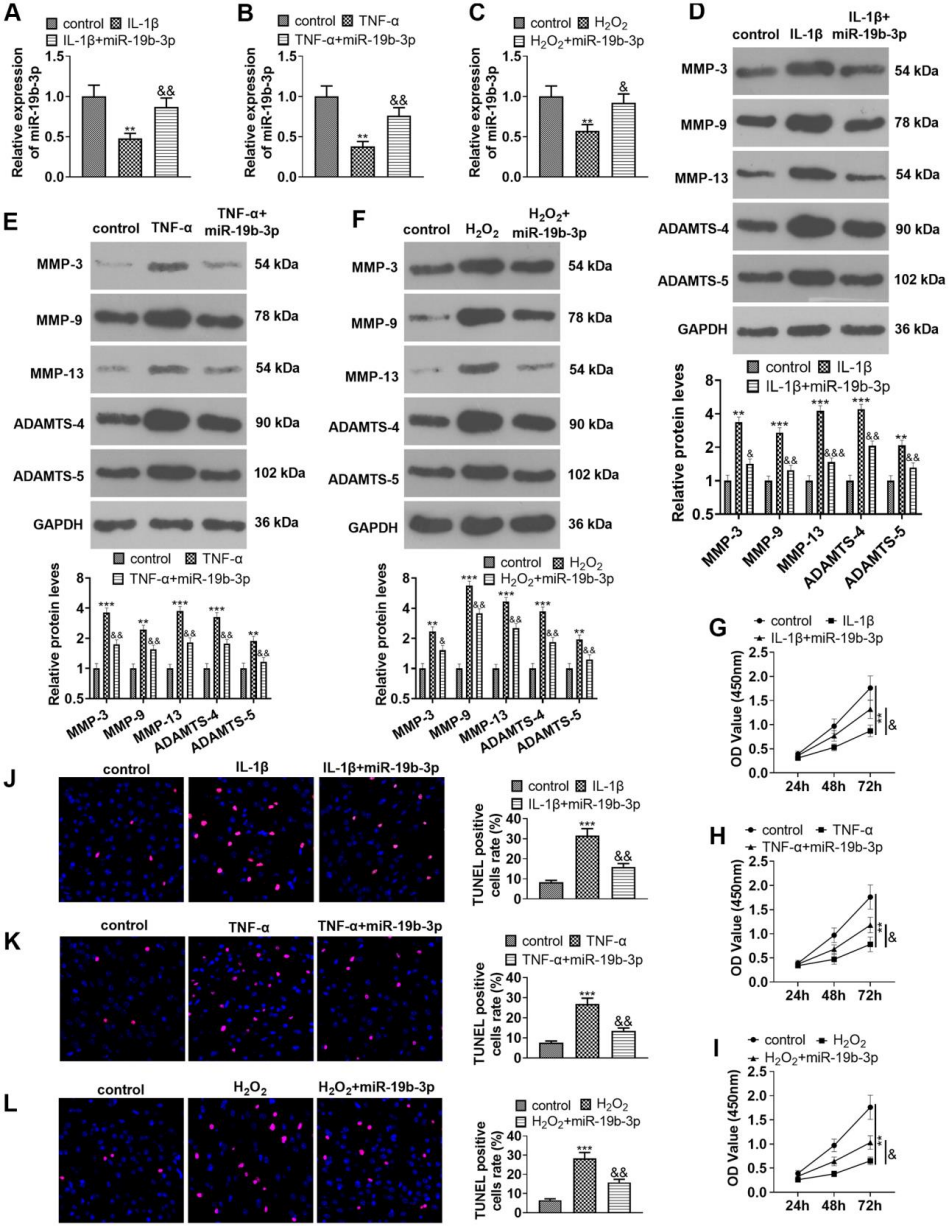
**Overexpressing *miR-19b-3p* attenuated IL-1β/TNF-α/hydrogen peroxide-mediated HNPC apoptosis.** In the *in-vitro* IVDD model, HNPCs were transfected with *miR-19b-3p* mimics. (**A**–**C**) The *miR-19b-3p* expression was determined by qRT-PCR. (**D**–**F**) The levels of MMP-3, MMP-9, MMP-13, ADAMTS-4 and ADAMTS-5 were compared by WB. (**G**–**I**) CCK-8 experiment was implemented to test cell proliferation. (**J**–**L**) Cell apoptosis was monitored by TUNEL staining ** *P* <0.01, *** *P* <0.001 (vs. control group). & *P* <0.05, && *P* <0.01, &&& *P* <0.001 (vs. the IL-1β/TNF-α/hydrogen peroxide group). N=3.

We transfected *miR-19b-3p* mimics into HNPCs treated with IL-1β, TNF-α, or hydrogen peroxide to probe into the effect of *miR-19b-3p* on HNPCs. qRT-PCR testified that the *miR-19b-3p* expression was dampened after the treatment of IL-1β, TNF-α, or hydrogen peroxide, while it was markedly up-regulated after overexpressing *miR-19b-3p* on this basis (vs. the IL-1β/TNF-α/hydrogen peroxide group) (*P*<0.05, Figure 3A–3C).

Additionally, the expression of MMP-3, MMP-9, MMP-13, ADAMTS-4 and ADAMTS-5 was examined with WB. Interestingly, their expression was distinctly reduced after cell transfection in comparison to that of the individual treatment of IL-1β, TNF-α, or hydrogen peroxide (*P*<0.05, Figure 3D–3F). Moreover, cell proliferation and apoptosis were verified by the CCK-8 assay (Figure 3G–3I) and TUNEL staining (Figure 3J–3L), respectively. The results confirmed that IL-1β, TNF-α or hydrogen peroxide dampened cell proliferation and enhanced apoptosis, while up-regulating *miR-19b-3p* on this basis facilitated cell proliferation and abated apoptosis (*P*<0.05, Figure 3G–3L). These outcomes concluded that *miR-19b-3p* overexpression repressed the IL-1β-, TNF-α-, or hydrogen peroxide-mediated HNPC apoptosis.

### The influence of overexpressing *miR-19b-3p* on PTEN/PI3K/Akt/mTOR pathway

**Figure 4:**
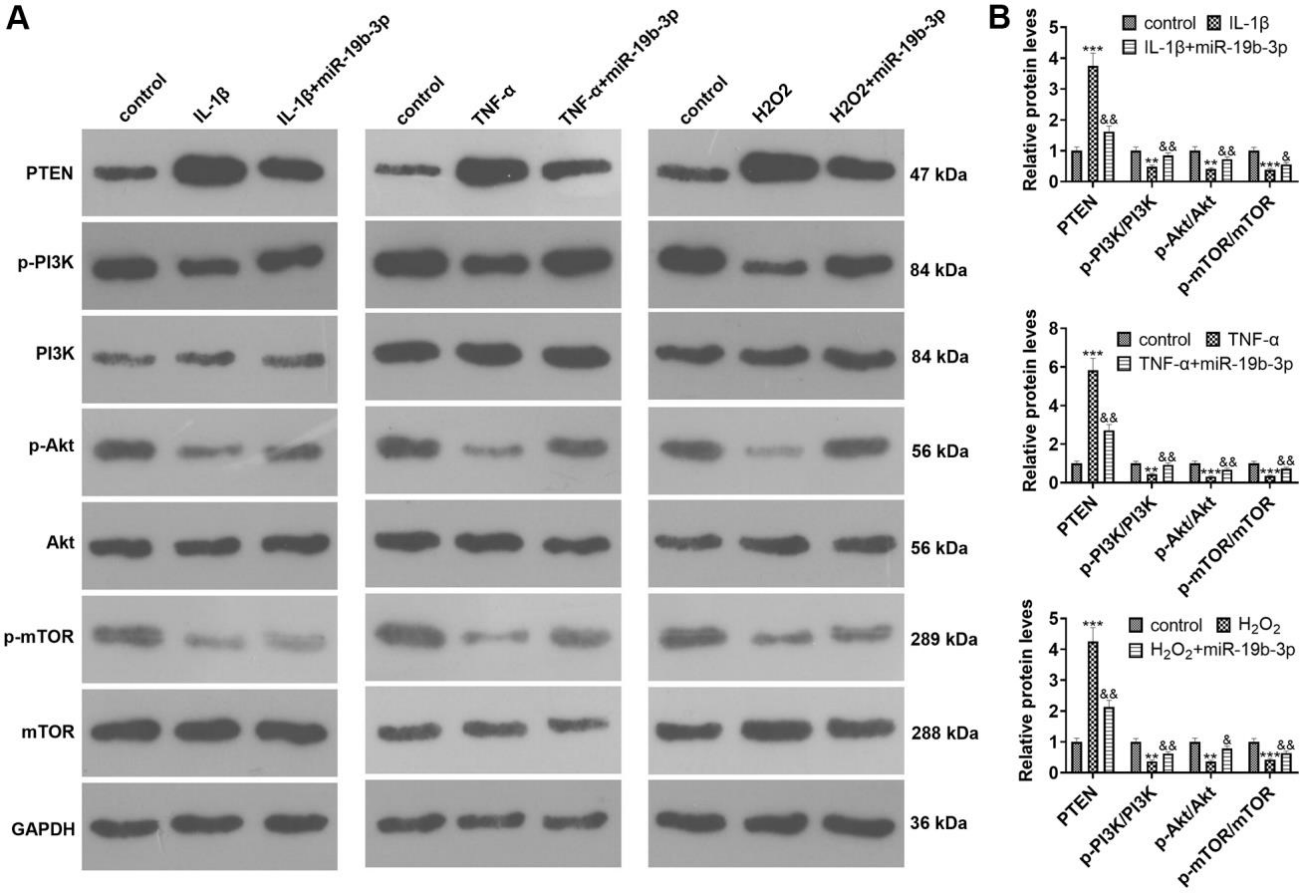
**The effect of overexpressing *miR-19b-3p* on the PTEN/PI3K/Akt/mTOR axis.** In the *in-vitro* IVDD model, HNPCs were transfected with *miR-19b-3p* mimics.. (**A**, **B**) WB was adopted to determine the PTEN/PI3K/Akt/mTOR expression. * *P* <0.05, ** *P* <0.01, *** *P* <0.001(vs. control group). & *P* <0.05, && *P* <0.01, &&& *P* <0.001(vs. the IL-1β/TNF-α/hydrogen peroxide group). N=3.

WB was implemented to clarify the impact of *miR-19b-3p* on PTEN/PI3K/Akt/mTOR pathway. The findings uncovered that the PTEN level was strengthened and the PI3K/Akt/mTOR signal was inactivated after IL-1β, TNF-α, or hydrogen peroxide treatment. In contrast, the PTEN level was elevated and the PI3K/Akt/mTOR axis was inactivated after cell transfection compared with that of the IL-1β/TNF-α/hydrogen peroxide group (*P*<0.05, Figure 4A, 4B).

### *miR-19b-3p* targeted the 3’UTR of PTEN and abated the PTEN expression

**Figure 5:**
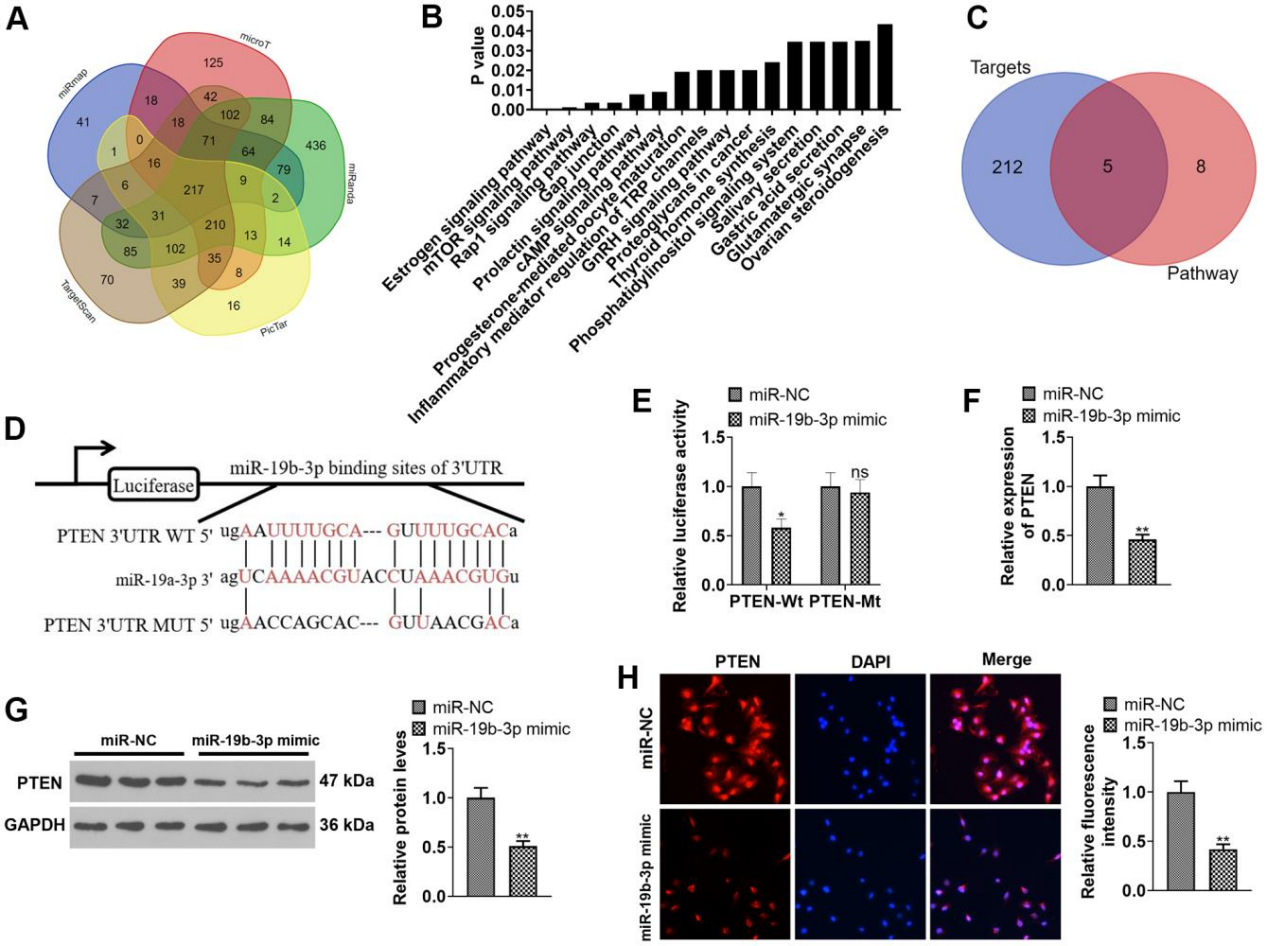
**Inhibiting PTEN enhanced the protective effect of *miR-19b-3p*.** (**A**) Detection of *miR-19b-3p* targets was made using the database ENCORI (http://starbase.sysu.edu.cn). (**B**) The database mirPath v.3 (http://snf-515788.vm.okeanos.grnet.gr) was applied to query the downstream pathway of *miR-19b-3p*. (**C**) Venn diagram of 217 targets of *miR-19b-3p* and the mTOR signaling pathway. (**D**) Base complementary sequences of *miR-19b-3p* and PTEN*.* 293-T cells were transfected with *miR-19b-3p* mimics for 24 hours. (**E**) The dual-luciferase reporter assay was implemented for ascertaining the binding relationship between *miR-19b-3p* and PTEN. HNPCs were transfected with *miR-19b-3p* mimics for 24 hours. (**F**–**H**) The PTEN profile in HNPCs was measured by qRT-PCR, WB and cellular immunofluorescence. ns*P*>0.05,* *P* <0.05, ** *P* <0.01 (vs.miR-NC group) N=3.

We adopted the database ENCORI (http://starbase.sysu.edu.cn) to predict the target gene of *miR-19b-3p*. As a result, *miR-19b-3p* has 217 common targets in miRmap, microT, miRanda, Pic Tar and Target Scan databases (Figure 5A). Moreover, through the mirPath v.3database (http://snf-515788.vm.okeanos.grnet.gr), we discovered that *miR-19b-3p* has 16 downstream pathways in the microT-CDS database (Figure 5B). As displayed in Figure 5C, the 217 targets of *miR-19b-3p* and the mTOR signaling pathway have five common target genes, including PTEN. Meanwhile, the ENCORI database revealed that *miR-19b-3p* targeted the 3’UTR of PTEN (Figure 5D). In addition, the dual-luciferase reporter assay ascertained that *miR-19b-3p mimics* attenuated the luciferase activity of PTEN-WT in 293T cells, but they exhibited no influence on PTEN-MUT (*P*<0.05, Figure 5E). By conducting qRT-PCR, WB and immunofluorescence, we observed that *miR-19b-3p* mimics notably choked the PTEN profile in HNPCs (*P*<0.05, Figure 5F–5H). Thus, *miR-19b-3p* targeted the 3 'UTR of PTEN and repressed the PTEN level.

### Inhibiting PTEN enhanced the protective function of *miR-19b-3p*

**Figure 6:**
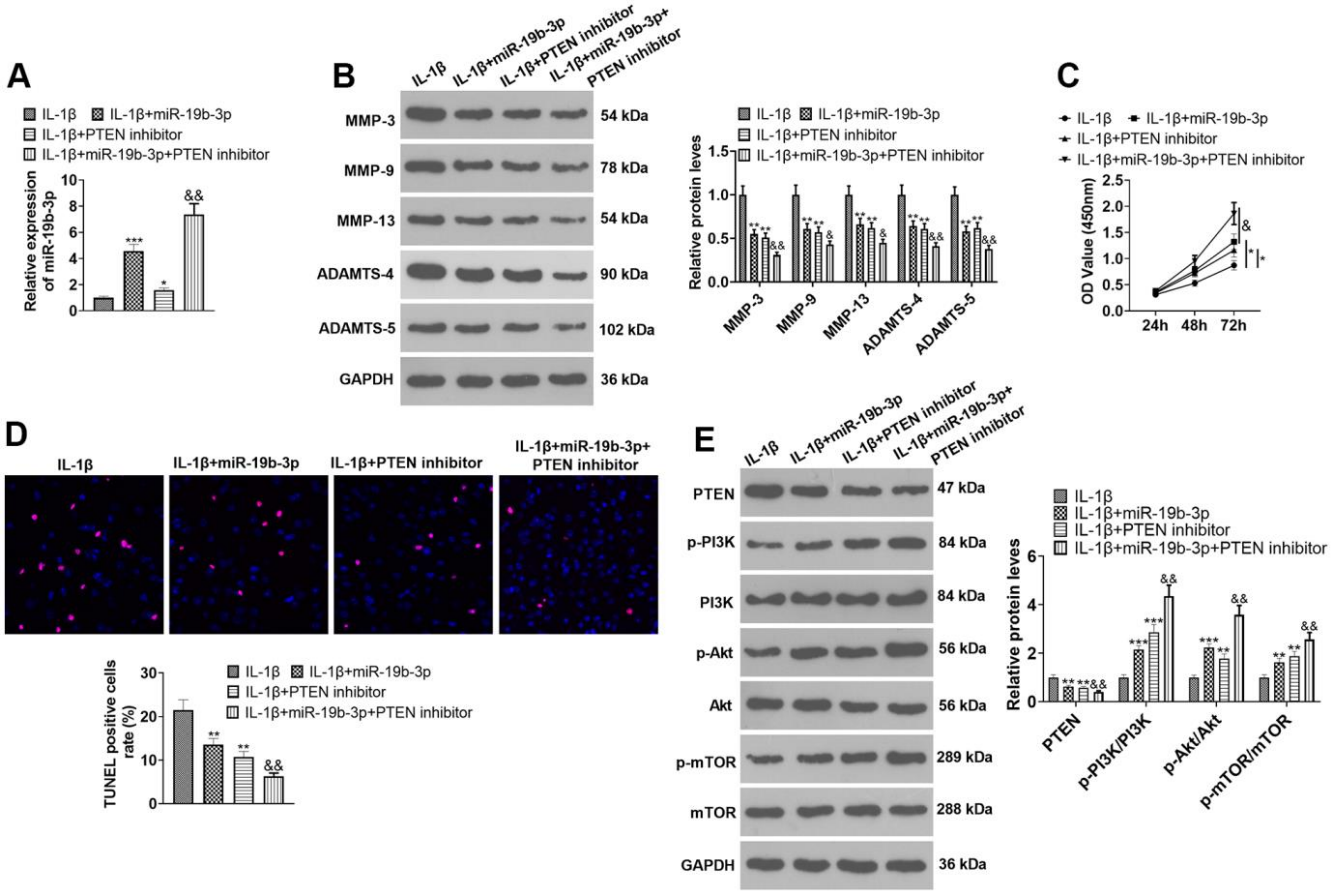
**Inhibiting PTEN enhanced the protective effect of *miR-19b-3p*.** In the *in-vitro* IL-1β-induced IVDD model, HNPCs were transfected with *miR-19b-3p* mimics or treated with the mTOR inhibitor (50 nM) for 24 hours. (**A**) The *miR-19b-3p* level was gauged by qRT-PCR. (**B**) The expression of MMP-3, MMP-9, MMP-13, ADAMTS-4 and ADAMTS-5 were tested by WB. (**C**, **D**) CCK-8 and TUNEL staining were adopted to examine cell proliferation and apoptosis of each group. (**E**) WB was used to test the PTEN/PI3K/Akt/mTOR pathway expression. * *P* <0.05, ** *P* <0.01, *** *P* <0.001 (vs.IL-1β group). & *P* <0.05, && *P* <0.01 (vs.IL-1β+*miR-19b-3p* group). N=3.

To confirm the potential role of miR-19b-3p-PTEN axis in HNPCs injury, IL-1β was used to construct an *in-vitro* model of IVDD. HNPCs were transfected with *miR-19b-3p* mimics or treated with the PTEN inhibitor. qRT-PCR outcomes demonstrated that both *miR-19b-3p* mimics and PTEN inhibitors enhanced the *miR-19b-3p* expression in HNPCs in comparison to the IL-1β group. Similarly, *miR-19b-3p* mimics elevated *miR-19b-3p* expression (vs. the IL-1β+PTEN inhibitor group) (*P*<0.05, Figure 6A). As displayed in Figure 6B, the PTEN inhibitor substantially curbed the levels of MMP-3, MMP-9, MMP-13, ADAMTS-4 and ADAMTS-5 compared with those of the IL-1β or IL-1β+*miR-19b-3p* groups (*P*<0.05). CCK-8 and TUNEL staining uncovered that the proliferative capacity of HNPCs was enhanced and the apoptotic capacity was diminished after the PTEN inhibitor treatment (*P*<0.05, Figure 6C, 6D). WB results displayed that the PTEN inhibitor distinctly abated the PTEN expression and strengthened the profiles of p-PI3K/PI3K, p-Akt/Akt and p-mTOR/mTOR (P<0.05, Figure 6E). These data revealed that inhibition of PTEN enhanced the protection of *miR-19b-3p* on HNPCs.

### Overexpressing *miR-19b-3p* weakened the attenuation of PTEN overexpression on HNPCs

**Figure 7:**
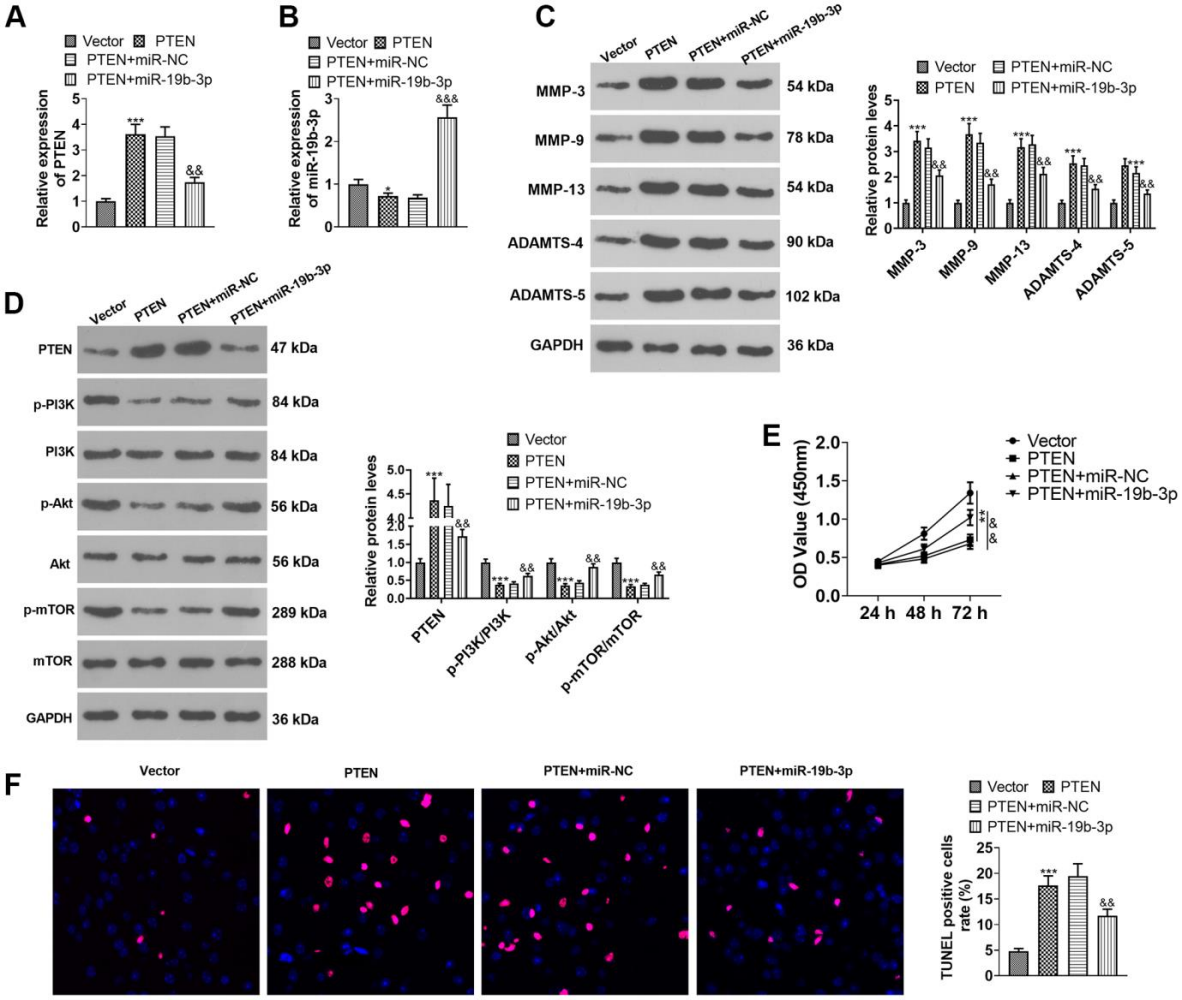
**Overexpressing *miR-19b-3p* attenuated the inhibition of PTEN overexpression on HNPCs.** HNPCs were transfected with PTEN overexpression plasmids or *miR-19b-3p* mimics. (**A**, **B**) The *miR-19b-3p* profile was checked by qRT-PCR. (**C**) WB was performed to compare the levels of MMP-3, MMP-9, MMP-13, ADAMTS-4 and ADAMTS- in HNPCs. (**D**) The PTEN/PI3K/Akt/mTOR expression tested by WB. (**E**) Cell proliferation was gauged by CCK-8. (**F**) TUNEL staining was performed to assess cell apoptosis. * *P* <0.05, ** *P* <0.01, *** *P* <0.001(vs. Vector group). & *P* <0.05,&& *P* <0.01(vs. Vector+miR-NC group). N=3.

HNPCs were transfected using PTEN overexpression plasmids or *miR-19b-3p* mimics. As displayed in Figure 7A, 7B, overexpression of PTEN repressed the *miR-19b-3p* expression (vs. the Vector group). Overexpressing *miR-19b-3p* hampered PTEN expression by contrast to the PTEN+miR-NC group (*P*<0.05). Overexpression of *miR-19b-3p* choked PTEN expression (vs. the PTEN+miR-NC group) (*P*<0.05). WB outcomes uncovered that overexpression of PTEN resulted in facilitated expression of MMP-3, MMP-9, MMP-13, ADAMTS-4, ADAMTS-5 and PTEN and declined expression of p-PI3K/PI3K, p-Akt/Akt and p-mTOR/mTOR. In contrast, compared to the PTEN+miR-NC group, overexpressing *miR-19b-3p* lowered the expression of MMP-3, MMP-9, MMP-13, ADAMTS-4, ADAMTS-5 and PTEN and boosted the expression of p-PI3K/PI3K, p-Akt/Akt and p-mTOR/mTOR (*P*<0.05, Figure 7C, 7D). As exhibited in Figure 7E, 7F, overexpressing *miR-19b-3p* reversed the anti-proliferative and pro-apoptotic effects of overexpression of PTEN on HNPCs. The above data disclosed that overexpressing *miR-19b-3p* attenuated the inhibitory effect of overexpressing PTEN on HNPCs.

### Inhibition of mTOR offset the protection of *miR-19b-3p*

**Figure 8:**
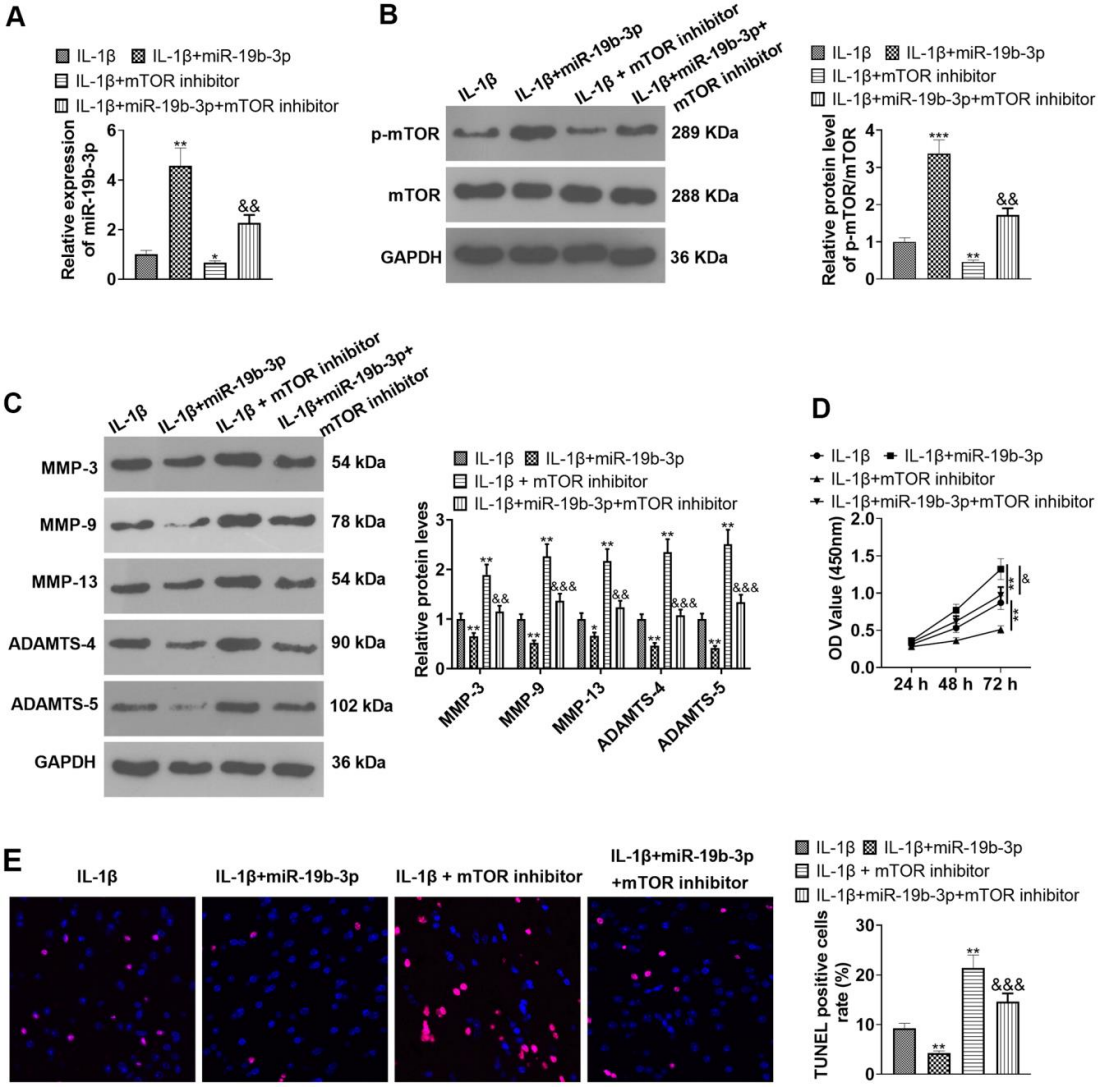
**Inhibition of mTOR offset the protective effect of *miR-19b-3p*.** In the *in-vitro* IL-1β induced IVDD model, HNPCs were transfected with *miR-19b-3p* mimics or treated with the mTOR inhibitor Rapamycin (0.1 nM) for 24 hours. (**A**) The *miR-19b-3p* expression was detected by qRT-PCR. (**B**) The phosphorylation level of mTOR was checked by WB. (**C**) WB was applied for assessing the PTEN/PI3K/Akt/mTOR pathway profile. (**D**) CCK-8 was employed to examine cell proliferation. (**E**) Cell apoptosis was evaluated by TUNEL staining. ** *P* <0.01, *** *P* <0.001 (vs.IL-1β group). & *P* <0.05, && *P* <0.01 (vs.IL-1β+*miR-19b-3p* group). N=3.

We conducted the following experiments to further clarify the role of mTOR in the process during which *miR-19b-3p* regulated HNPCs. First, HNPCs were treated with IL-1β. Then, they were transfected with *miR-19b-3p* mimics and treated with the mTOR inhibitor torin1. qRT-PCR verified that *miR-19b-3p* expression was notably hindered by the mTOR inhibitor compared with the IL-1β or IL-1β+*miR-19b-3p* group (*P*<0.05, Figure 8A)*.* WB outcomes uncovered that by contrast with the IL-1β or IL-1β+*miR-19b-3p* groups, the mTOR inhibitor obviously attenuated the p-mTOR/mTOR level in HNPCs (*P*<0.05, Figure 8B). Besides, the profiles of matrix metalloproteinases MMP-3, MMP-9, MMP-13, ADAMTS-4 and ADAMTS-5 were strongly inhibited by the mTOR inhibitor (*P*<0.05, Figure 8C). CCK-8 and TUNEL staining outcomes validated that HNPC proliferation was abated and apoptosis was enhanced after the mTOR inhibitor treatment compared with the IL-1β or IL-1β+*miR-19b-3p* groups (*P*<0.05, Figure 8D, 8E). In conclusion, overexpressing *miR-19b-3p* facilitated cell proliferation and dampened cell apoptosis, while the inhibition of mTOR offset the protective effect of *miR-19b-3p*.

## DISCUSSION

IVD is the largest avascular organ in the human body [19], and the incidence of IVD disease increases proportionally with the aging of the population [20]. However, the current conservative treatment and surgical treatment have limited effects, which can only relieve symptoms and cannot restore the structural and biological function of the IVD [21]. Here, we discovered that overexpressing *miR-19b-3p* attenuates IL-1β-, TNF-α-, hydrogen peroxide-mediated HNPC apoptosis. Further studies displayed that *miR-19b-3p* targeted the 3’UTR of PTEN and choked PTEN expression, thus activating PI3K/Akt/mTOR pathway.

Multiple pieces of research have confirmed that miRNAs contribute to regulating IVDD. It is reported that *MIR660* knockout exerts a protective role in the apoptosis of NPs by up-regulating SAA1mRNA [22]. Other scholars have claimed that *miR-499a-5p* reduces TNF-α-induced HNPC apoptosis and the imbalance between anabolism and catabolism of extracellular matrix by down-regulating SOX4 [23]. On the other hand, *miR-19b-3p* studied in this paper is a miRNA involved in regulating cell inflammation and metabolic-related diseases. For example, H19 overexpression dampens BMSC proliferation and differentiation by hampering *miR-19b-3p* expression in postmenopausal OP [7]. In addition, Xu H et al. discovered that the *miR-19b-3p* level in sepsis patients’ serum is notably reduced and is reversely linked to the IL-6 and TNF-α contents. More importantly, *miR-19b-3p* facilitation mitigates sepsis-induced inflammation [24]. Here, it was revealed that *miR-19b-3p* is down-regulated in the tissues of IVDD patients and HNPCs treated with IL-1β, TNF-α, or hydrogen peroxide. Interestingly, overexpressing *miR-19b-3p* attenuates the anti-proliferative and pro-apoptotic effects-mediated by IL-1β, TNF-α, and hydrogen peroxide on HNPCs, suggesting that miR-19b-3p serves as a potential therapeutic target against IVDD.

PTEN, a homolog of phosphatase and tension protein missing from chromosome 10, is an effective tumor suppressor that regulates cell growth and survival [25, 26]. For instance, *miR-221* has been reported to curb PTEN expression and activate phosphatidylinositol 3 kinase (PI3K)/AKT, thus repressing the apoptosis of cardiomyocytes (CMs) [27]. In addition, Per2 facilitates NHAC-kn inflammation in osteoarthritis by heightening the PTEN level and dampening the PI3K/Akt expression [28]. Of special note, PTEN is overexpressed in denatured NP. Moreover, VO-OHpic (VO), a PTEN inhibitor, can prevent NP degradation by diminishing oxidative stress and increasing cell proliferation through the PTEN/Akt pathway [29]. In our study, qRT-PCR and WB confirmed that PTEN is up-regulated in the tissues of IVDD patients and HNPCs treated with IL-1β, TNF-α, or hydrogen peroxide. Furthermore, the functional assays confirmed that PTEN inhibition relieved HNPCs injury, and *miR-19b-3p* exert protective effects on HNPCs via targeting PTEN.

The mammalian target of rapamycin (mTOR) is a vital mediator of the PTEN/PI3K/AKT signaling [30]. mTOR signals control basic biological functions, including proliferation, growth, metabolism, autophagy, and aging, and its excessive activation leads to various human diseases [31]. For example, the down-regulation of *miR-181* dampens the PTEN level and induces the expression of PI3K, P-Akt, and p-mTOR, confirming that knockdown of *miR-181* strengthens the proliferation and migration of A549/DDP cells [32]. On the other hand, resveratrol (RSV) boosts cell viability and impedes apoptosis by motivating the *miR-17*-regulated PTEN/PI3K/AKT and mTOR pathways and hampers inflammatory damage of HaCaT cells by reducing the production of IL-6/IL-8/TNF-α [33]. Above all, osteoblasts protein 1 (OP-1) dampens the apoptosis of rat NPs by activating PI3K/Akt/mTOR in hypertonic cultures [34]. In contrast, another study revealed that the PI3K/Akt phosphorylation protects IDD, ascribing to increased ECM concentrations, apoptosis prevention, cell proliferation elevation, induction or repression of autophagy, reduction of oxidative damage and adaptive hypoxic microenvironment [35]. Here, we discovered that inhibition of mTOR offsets the protection of *miR-19b-3p* on HNPCs. Overall, through a series of experiments, it was discovered that *miR-19b-3p* is down-regulated in the tissues of IVDD patients and HNPCs. Meanwhile, *miR-19b-3p* overexpression motivates PI3K/Akt/mTOR by hampering PTEN, thus heightening HNPC proliferation and choking its apoptosis, which provides new insights and theoretical references for the treatment and intervention of IVDD patients clinically.

## MATERIALS AND METHODS

### Collection and treatment of clinical specimens

The IVD tissues from 20 IVDD patients who received surgical treatment in Qilu Hospital of Shandong University (Qingdao), and the normal IVD tissues removed from 10 patients due to trauma from January 2019 to May 2019 were harvested. All patients signed the informed consent, and the experiment was authorized by the Ethics Committee of Qilu Hospital of Shandong University (Qingdao) and met the ethical requirements of international and national regulatory authorities for biomedical research. All the patients were diagnosed with IVDD by two senior pathologists, and the tissues were placed in liquid nitrogen at -196° C for future use.

### Cell culture

Human NP cells (HNPCs) were isolated from the normal IVD tissues. In short, the tissues were cut into sections of about 1 mm^3^ and treated for 0.5 hours with 0.25% trypsin (Gibco, Life Technologies, Paisley, UK), and then digested for 3 hours with 0.2% type II collagenase (Invitrogen, Carlsbad, CA, USA) at 37° C. After filtration and washing with PBS, the suspension was centrifuged, and the cells were cultured in the Dulbecco modified Eagle medium comprising F12 nutrient mixtures (Gibco, Grand Island, NY, USA), 15% fetal bovine serum (FBS; Invitrogen, Carlsbad, CA, USA) and 1% penicillin/streptomycin (Sigma-Aldrich, St.Louis, MO, USA) at 37° C with 5% CO_2_. After fusion, HNPCs were digested and sub-cultured. The second-generation cells were applied for subsequent experiments.

An *in-vitro* model of IVDD was constructed by treating HNPCs with 10 ng/mL IL-1β (Novoprotein, Shanghai, China), 40 ng/mL TNF-α (PeproTech, East Windsor, NJ, USA), 500 μM hydrogen peroxide (H_2_O_2_) (349887, Sigma-Aldrich, MO) for 24 hours, respectively. Then, the cells were harvested for later experiments.

### Cell transfection

HNPCs at the logarithmic growth stage were seeded in 6-well plates at 5×10^6^/well after digestion and sub-culture. After stable cell growth, HNPCs were transfected with *miR-19b-3p* mimics and corresponding negative control group (miR-NC), PTEN overexpression plasmids and Vectors, according to the specification of the FuGENE®HD Transfection Reagent (Roche, Shanghai, China). At last, they were maintained at 37° C with 5% CO_2_ for 24 hours.

### Quantitative real-time polymerase chain reaction (qRT-PCR)

Total RNAs were separated from IVD tissues or HNPCs with the TRIzol reagent (Invitrogen, Carlsbad, CA, USA). After the RNA concentration and purity were tested by utilizing Thermo NanoDrop 2000, the miRNA and mRNA were reversely transcribed into cDNA by adopting the One Step PrimeScript miRNA cDNA synthesis kit (Bao Biological Engineering Co., Ltd., Dalian, China) and RevertAid First Strand cDNA short Kit (Thermo Fisher Scientific, Waltham, MA, USA) respectively. The cDNA synthesis was performed at 37° C for 40 min and 85° C for 5 s. PCR amplification was implemented with the SYBRGreen method and cDNA as template, while *miR-19b-3p*, MMP-3, MMP-9, MMP-13, ADAMTS-4 and ADAMTS-5 served as specific primers (Sangon Biotech, Shanghai, China). PCR was performed with 40 cycles of 95° C for 30 s, 95° C for 5 s, 60° C for 30 s and 73° C for 10s. *U6* was the endogenous control of *miR-19b-3p*, while *GAPDH* was that of *MMP-3, MMP-9, MMP-13, ADAMTS-4* and *ADAMTS-5*. The relative expression was measured with the 2 ^- Δ Δ CT^ method, and the primer sequences were exhibited in Table 1.

**Table 1:** The primers used in qRT-PCR.

**Gene name**	**Primer sequence (5’→3’)**
*miR-19b-3p*	F:CACTGTTCTATGGTTAG
	R:CACTACCACAGTCAGTT
*MMP-3*	F:GCTGTTTTTGAAGAATTTGGGTTC
	R:GCACAGGCAGGAGAAAACGA
*MMP-9*	F:CTTTGAGTCCGGTGGACGAT
	R:TCGCCAGTACTTCCCATCCT
*MMP-13*	F:ATGCAGTCTTTCTTCGGCTTAG,
	R:ATGCCATCGTGAAGTCTGGT
*ADAMTS-4*	F:ACTGGTGGTGGCAGATGACA
	R:TCACTGTTAGCAGGTAGCGCTTT
*ADAMTS-5*	F:GCTTCTATCGGGGCACAGT
	R:CAGCAGTGGCTTTAGGGTGTAG
*PTEN*	F:AGTTCCACCCCTTCCATCTG
	R:ACCGGCAGCATCAAATGTTT
*GAPDH*	F:CTCCTCCTGTTCGACAGTCAGC
	R:CCCAATACGACCAAATCCGTT
*U6*	F:GACGAAGAGGATTCGCTGAC
	R:AAATCTAGCTGCTGCGGTTC

### Western blot (WB)

After the HNPCs were treated with varying factors, the primary medium was removed. Then, the cells were lysed with RIPA (Beyotime, Shanghai, China) lysis buffer (containing 1% PMSF) and harvested through centrifugation (14000 rpm for 30 min at 4° C), and the total cellular protein was extracted. Proteins were quantified using the Bradford method, and the samples were denatured by boiling in 100° C water for 5 min and then centrifuged for 30 s after ice-cooling. Afterward, the supernatant was taken for polyacrylamide gel electrophoresis and transferred to polyvinylidene fluoride (PVDF) membranes (Millipore Corporation, Bedford, MA, USA) under 100 V for 1 hour. After being blocked with 5% skimmed milk at room temperature (RT) for 1 hour, the membranes were incubated overnight at 4° C with the primary Anti-MMP-3 antibody (1:2000, ab52915), Anti-MMP-9 antibody (1:2000, ab76003), Anti-MMP-13 antibody (1:4000, ab39012), Anti-ADAMTS-4 antibody (1:1000, ab185722), Anti-ADAMTS-5 antibody (1:500, ab41037), Anti-PTEN antibody (1:1000, ab267787), Anti-PI3K antibody (1:1000, ab191606), Anti-p-PI3K antibody (1:1000, ab182651), Anti-p-Akt antibody (1:1000, ab38449), Anti-Akt antibody (1:500, ab8805), Anti-mTOR antibody (1:1000, ab32028) and Anti-GAPDH antibody (1:1000, ab8245) from Abcam (Waltham, MA, USA), and Anti-p-mTOR (pSer2448) antibody (1:1000, SAB4504476) from Sigma-Aldrich (Shanghai, China). Next, the membranes were rinsed with TBST twice and maintained with horseradish peroxidase (HRP)-labeled Goat Anti-Rabbit (1:2500, AB6721) for 1 hour at RT. After being rinsed three times, the membranes were exposed with ECL chromogenic agents and developed with a membrane scanner.

### Cell counting kit-8 (CCK-8) experiment

HNPCs were digested and collected, then the single cell suspension was made (2×10^3^/mL). Then, they were dispersed in 96-well plates with 100 μL cell suspension per well, and each group had three repetitive wells. Next, the plates were placed in an incubator. After 24 hours, 10 μL CCK-8 solution (Hubei Biossci Biotechnology Co., Ltd, China) was added to each well for incubation for another 1 hour. After culture, the plates were put in a microplate reader to observe the optical density (OD) of each well at 450 nm. Afterward, the OD of each cell was measured at the 24^th^, 48^th^ and 72^ed^ hour, respectively.

### TdT-mediated dUTP nick end labeling (TUNEL) assay

HNPCs treated with the different factors were seeded into 24-well plates (1 × 10^5^ per well) and further maintained for 24 hours. Cells were fastened with 4% paraformaldehyde for 30 min and cleaned with PBS. 0.2% DE Triton X-100 was added and incubated with the cells at RT for 15 min. Next, cell apoptosis was examined by employing the TUNEL apoptosis detection kit (Shanghai Xinyu Biotech. Co., Ltd, Shanghai, China). Briefly, 50 μL of TUNEL reaction solution was added and incubated at RT away from light for 1 hour. Then, DAPI (Shanghai Biotime Biotechnology Co., Ltd, Shanghai, China.) was applied for re-staining for 1 min. Cells were immersed in PBS and sealed with the anti-fade mounting medium. Five randomly chosen non-overlapping fields of view were reviewed with a microscope, and the TUNEL-positive cells were counted.

### Dual-luciferase reporter gene assay

The bioinformatics predicted that *miR-19b-3p* targeted 3’-UTR of PTEN. All luciferase reporter vectors (PTEN-WT and PTEN-MUT) were constructed by Promega (Promega, Madison, WI, USA). 293T cells (4.5×10^4^) were dispersed in 48-well plates and cultured to reach a 70% confluence rate. Then, PTEN-WT and PTEN-MUT were co-transfected with *miR-19b-3p* mimics or negative controls, respectively, using liposome 2000. After the transfection for 48 hours, the luciferase activity was determined as requested by the manufacture. All tests were made in triplicate.

### Cellular immunofluorescence

HNPCs were inoculated into 24-well plates (1 × 10^5^ cells/well) and transfected with *miR-19b-3p* and miR-NC, respectively, for 48 hours. Cells were immobilized with 4% paraformaldehyde at RT for 20 min and endogenous peroxidase was inactivated by the addition of 3% H_2_O_2_ for 15 min. Cells were subjected to blocking with 5% goat serum for 1 hour and incubation with the primary PTEN antibody (1:80, ab170941, Abcam) overnight at 4° C. The following morning, a secondary antibody, Goat Anti-Rabbit (1:100, ab6721, Abcam), was added dropwise and maintained for one hour at RT. DAPI staining solution was utilized for cell dyeing for 5 min at RT away from light. After mounting with the anti-fade mounting medium, the cells were reviewed and photographed under a fluorescence microscope.

### Statistical analysis

Experimental data were processed with the SPSS18.0 statistical software (SPSS Inc., Chicago, IL, USA) and GraphPad Prism 8 (GraphPad Software, USA). The differences between the two groups were ascertained by the *t* test, while one-way ANOVA was employed to analyze differences between multiple groups. *P* <0.05 indicated statistical significance. Pearson correlation coefficient R was adopted to measure the correlation between *miR-19b-3p* and PTEN in the IVD tissues of IVDD patients. Quantitative data were presented as mean values and SD values of independent experiments that were repeated at least three times.

### Ethics statement

Our study was approved by the Ethics Review Board of Qilu Hospital of Shandong University (Qingdao).

### Data availability

The data sets used and analyzed during the current study are available from the corresponding author on reasonable request.
